# 

**Published:** 1847-06

**Authors:** 


JUNE AND JULY, 1847.

VOL. II. NEW SERIES. NO. 2.

### ILLINOIS AND INDIANA MEDICAL AND SURGICAL JOURNAL.

PUBLISHED EVERY OTHER MONTH.

EACH NUMBER CONTAINING NINETY-SIX PAGES.

EDITED BY

JAMES V. Z. BLANEY, M. D., DANIEL BRAINARD, M. D., WM. B. HERRICK, M. D., AND JOHN EVANS, M. D.

PROFESSORS IN RUSH MEDICAL COLLEGE, CHICAGO, ILLINOIS.

INDIANAPOLIS, IND.:
PUBLISHED BY JOHN D. DEFREES.

CHICAGO, ILL.:
PUBLISHED BY WILLIAM ELLIS.

1847.

TWO DOLLARS PER ANNUM, IN ADVANCE.

